# Recent Advances on Extracellular Vesicles: A Natural Nanomaterial for Biomedical Application

**DOI:** 10.3390/biomimetics11060416

**Published:** 2026-06-11

**Authors:** Fan Li, Siyu Liu, Shuaiwei Xu, Huimin Duan, Yanchao Wang, Jingan Li

**Affiliations:** 1 School of Materials Science and Engineering, Zhengzhou University, Zhengzhou 450001, China; lifan349@gs.zzu.edu.cn (F.L.); liu_siyu@gs.zzu.edu.cn (S.L.); duanhuimin@gs.zzu.edu.cn (H.D.); wangyancaho@gs.zzu.edu.cn (Y.W.); 2 School of Materials Electronics and Energy Storage, Zhongyuan University of Technology, Zhengzhou 451191, China; 2024105026@zut.edu.cn

**Keywords:** extracellular vesicles (EVs), exosomes, engineering transformation, drug delivery systems, clinical translation

## Abstract

Extracellular vesicles (EVs), naturally secreted by cells as nanoscale lipid bilayer structures, have become a research hotspot in biomedicine owing to their excellent biocompatibility, low immunogenicity, and inherent ability to cross biological barriers. This review systematically summarizes recent advances in EVs as natural nanomaterials. The biogenesis mechanisms of EVs are outlined, followed by a comparative analysis of the advantages and limitations of mainstream isolation and purification methods, including ultracentrifugation, size-exclusion chromatography, and microfluidic technologies. The core guiding role of the MISEV 2023 guidelines in standardizing EV characterization is highlighted. Engineering strategies to enhance EV therapeutic efficacy—including parental cell modification, post-isolation physicochemical tailoring, and hybrid vesicle construction—are then reviewed, followed by a comparative analysis of mainstream isolation technologies, emphasizing the trade-offs between purity and yield. Distinct from conventional descriptive reviews, this article establishes a strong biomimetic framework to scrutinize engineering strategies, including parental cell genetic modification, post-isolation physicochemical tailoring, and the fabrication of hybrid bio-synthetic vesicles. The design principles governing targeted delivery, drug-loading physics, and in vivo pharmacokinetic stability are critically evaluated through the lens of biomimetic nanotechnology. Furthermore, we identify critical research gaps and technical bottlenecks impeding clinical translation, offering a forward-looking perspective on the evolution of EVs from natural messengers into standardized precision medicine platforms.

## 1. Introduction

**Figure 1:**
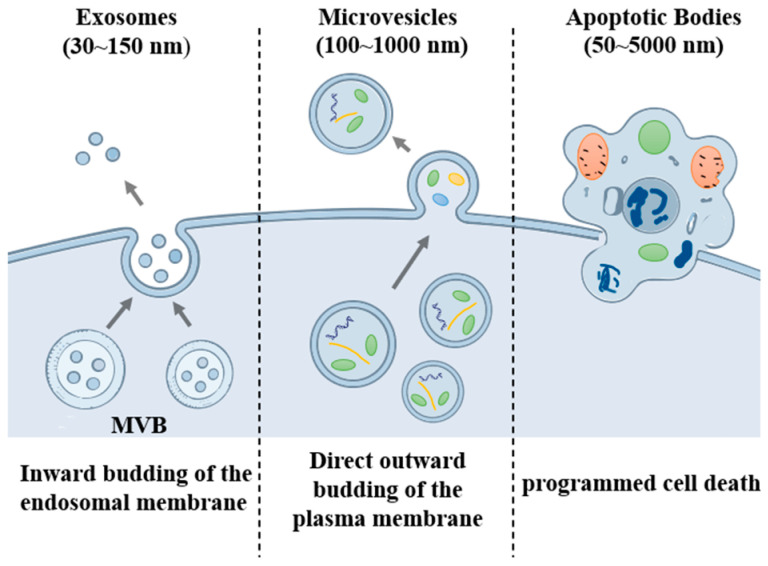
Schematic overview of EV biogenesis.

Extracellular vesicles (EVs) are heterogeneous, nanosized, membrane-enclosed structures released by nearly all cell types [1,2,3,4]. They can be broadly classified based on their biogenesis and physical dimensions. Major subtypes include exosomes (30–150 nm), microvesicles (100–1000 nm), and apoptotic bodies (50–5000 nm) [3,4,5,6]. However, precise categorization remains challenging due to overlapping size ranges and shared biogenetic pathways. Exosomes originate from the inward budding of the endosomal membrane, leading to the formation of intraluminal vesicles within multivesicular bodies (MVBs), and subsequent fusion of MVBs with the plasma membrane results in their release [7,8]. In contrast, microvesicles are generated by direct outward budding of the plasma membrane [4,9]. Apoptotic bodies are larger vesicles released during programmed cell death [10] (Figure 1).

EV biogenesis involves diverse molecular mechanisms. For exosomes, the endosomal sorting complexes required for transport (ESCRT) system and non-ESCRT pathways play key roles. Microvesicle formation is driven by cytoskeletal rearrangements and membrane budding regulators (e.g., ARF6, Rho GTPases), while apoptotic body release occurs during programmed cell death through distinct caspase-dependent mechanisms [11,12,13,14]. EVs encapsulate a diverse array of biomolecules derived from their parent cells, such as proteins (e.g., ALIX, TSG101, flotillin-1, syntenin-1, and tetraspanins including CD9, CD63, and CD81), nucleic acids (mRNA, miRNA, DNA), lipids (e.g., phosphatidylserine, phosphatidic acid, sphingomyelin), and metabolites [15,16,17,18,19]. The specific molecular cargo reflects the physiological and pathological state of the parental cell [20,21,22,23,24].

**Figure 2:**
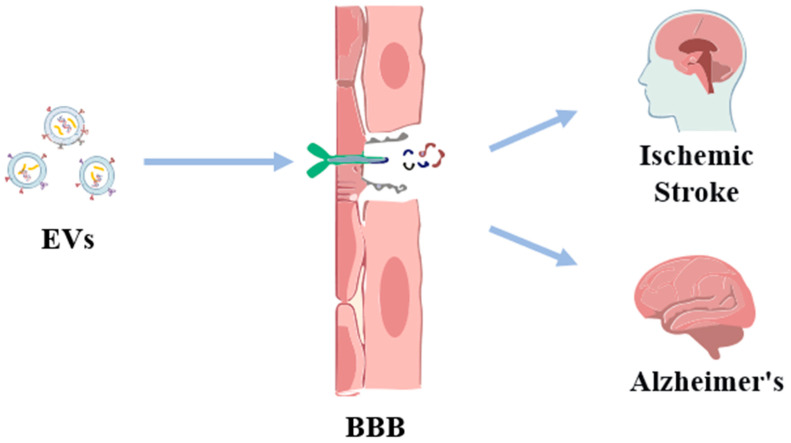
EVs Cross the BBB for Neurological Disease Therapy. Engineered EVs can traverse the BBB via receptor-mediated transcytosis, delivering therapeutic cargo to the brain parenchyma.

Early studies once regarded EVs as cellular “waste products” generated during metabolism. However, this perception has fundamentally shifted over the past two decades, with EVs now redefined as key messengers mediating intercellular communication over both short and long distances and playing central roles in processes such as immunomodulation, tumor microenvironment remodeling, and tissue repair [25,26,27,28,29,30,31]. In recent years, with the rapid advancement of nanotechnology and molecular biology, the unique advantages of EVs as natural drug carriers have become increasingly evident: they are derived from autologous or allogeneic cells, exhibiting low immunogenicity and high biocompatibility; their lipid bilayer structure protects the encapsulated cargo from enzymatic degradation in vivo; and importantly, some EV subtypes have been shown to possess the ability to traverse physiological barriers such as the blood–brain barrier (BBB) (Figure 2), albeit with variable efficiency depending on their cellular origin, surface composition, and isolation methods [32,33,34,35,36]. These characteristics have positioned EVs as a focal point in drug delivery system research, with hundreds of EVs-related clinical trials currently registered worldwide, spanning multiple fields including oncology, neurological disorders, and tissue regeneration [37].

Despite the numerous advantages of natural EVs, their clinical application remains hindered by limitations such as insufficient targeting capacity, low drug loading efficiency, and rapid in vivo clearance [38,39]. Consequently, enhancing EV functionality through engineering strategies has emerged as a central focus of current research. This review summarizes the progress of engineered EVs in disease treatment. It begins with an overview of EV fundamentals and isolation and characterization techniques, then focuses on the main engineering strategies, including parental cell engineering, direct post-isolation modification, and the construction of hybrid EVs. Subsequently, it reviews preclinical advancements in the application of engineered EVs for tumor therapy, central nervous system disorder intervention, and tissue repair. Finally, the challenges facing clinical translation and future directions are discussed, aiming to provide insights for research in related fields.

In this review, we regard EVs not merely as intercellular messengers, but as highly complex natural nanocarriers. Their modular structural organization epitomizes the sophisticated design principles shaped by evolution. The unique and core scientific contribution of this review lies in constructing an integrated biomimetic framework that seamlessly bridges the fundamental biology of EVs and advanced nanobiotechnology. By deeply dissecting the roles of their native lipid bilayer composition, transmembrane topology, and surface glycans in mediating immune evasion and traversing robust biological barriers such as the blood–brain barrier, this review extracts a precise design blueprint that can be leveraged by materials science researchers, thereby inspiring the rational engineering of synthetic biomimetic nanoparticles. Accordingly, this review is not a mere compilation of empirical data, but rather focuses on critically analyzing how to learn from and repurpose nature’s design principles to precisely overcome the inherent technical limitations of artificial drug delivery platforms.

## 2. Isolation, Characterization, and Standardization of EVs

Before engineering EVs for therapeutic applications, it is essential to establish robust methods for their isolation and characterization, as the purity and integrity of starting material directly impact the success of downstream engineering.

### 2.1. Main Isolation Methods and Their Principles

The isolation and purification of EVs forms the foundation of EV research, with the core objective of obtaining a population of highly pure and structurally intact vesicles. Currently, the mainstream isolation methods include the following:

As the most widely used method for isolating extracellular vesicles, differential ultracentrifugation (UC) is based on progressively increasing centrifugal forces and employs a multi-step centrifugation process to sequentially remove cell debris and subcellular components from the sample [40]. The advantage of UC lies in its relatively straightforward procedure and its suitability for processing large-volume samples. However, its significant limitations include the time-consuming nature of the procedure and the potential damage to vesicular structural integrity caused by mechanical shear forces generated during repeated centrifugation cycles [41].

Size exclusion chromatography (SEC) operates on the principle of molecular sieving, utilizing the size-dependent separation effect of a porous gel medium to achieve precise isolation based on particle size [40,42]. Vesicles with larger diameters cannot enter the gel pores and are eluted first, while smaller impurities are retained within the column, resulting in longer retention times. This strategy employs mild conditions, maximally preserving the native morphology and structural integrity of the vesicles, which renders it particularly suitable for subsequent functional studies [41]. However, in complex biological samples such as plasma, the purity of EVs obtained by SEC is often relatively limited, primarily due to the co-elution of impurity components such as lipoproteins and protein aggregates [43]. Furthermore, this isolation process frequently requires additional sample concentration steps to meet the demands of subsequent analysis or applications.

Immunoaffinity capture (IAC) leverages the high affinity of antibodies for specific surface markers on vesicles (e.g., CD9, CD63, CD81) to selectively enrich particular subpopulations from complex biological samples [44,45]. This method offers excellent selectivity, enabling precise identification of target vesicles [46]. Nevertheless, its yield is often limited, the cost is relatively high, and the binding of antibodies to surface markers may potentially interfere with the vesicles’ native conformation and subsequent functional analyses [47]. Moreover, this approach may introduce selection bias toward specific EV subpopulations expressing the targeted marker, potentially excluding functionally important vesicles lacking that marker, and its poor scalability and high cost present substantial hurdles for industrial-scale manufacturing [48].

Polymer precipitation (PP) methods involve the introduction of hydrophilic polymers such as polyethylene glycol to alter the solubility of vesicles in solution, thereby promoting their sedimentation and enrichment [40]. This approach is simple and rapid, making it suitable for the initial concentration of large-volume samples, and most commercially available kits are developed based on this principle [49]. However, due to the non-specific nature of the precipitation mechanism, it is prone to co-precipitation of impurities such as lipoproteins and protein aggregates, which can affect purity.

As an innovative platform that has emerged in recent years, microfluidic (MF) technology integrates multiple strategies including immunoaffinity capture, size-based sorting, and acoustic manipulation to achieve rapid and precise isolation of extracellular vesicles [46,47]. MF platforms integrate multiple separation mechanisms—such as immunoaffinity capture, size-based sorting, and acoustic or dielectrophoretic manipulation—within a miniaturized chip, enabling rapid and precise EV isolation from small sample volumes. This technology offers significant advantages such as low sample consumption, high integration, and flexible operation. However, it remains in the early stages of development, and the standardization of its protocols and pathways for large-scale production still requires further investigation and harmonization [50,51].

It is essential to emphasize that each method has its own advantages and limitations. Researchers should select the appropriate method or a combination of methods based on the requirements of subsequent experiments, and clearly describe the isolation protocol when reporting results. The differences and distinctions between different methods are summarized in Table 1.

### 2.2. Characterization Techniques for EVs

The characterization of extracellular vesicles requires the orthogonal validation of multiple parameters, and confirmation of their vesicular identity and purity can only be achieved through comprehensive analysis across multiple dimensions, including particle size, morphology, and markers. Below we describe selected examples of commonly used approaches, which are not intended to be exhaustive.

Particle size analysis constitutes a fundamental experiment in EV characterization. Nanoparticle tracking analysis (NTA) calculates particle size distribution and concentration by measuring Brownian motion trajectories, enabling rapid assessment of particle size distribution and concentration, and is currently the most commonly employed method for particle size determination [52]. Although dynamic light scattering (DLS) can also measure particle size, it is more sensitive to larger particles and tends to underestimate the proportion of small EVs; therefore, its application in complex samples requires caution [53].

Morphological observation allows for the direct visualization of vesicle integrity. Transmission electron microscopy (TEM) can be used to observe the overall morphological characteristics of EVs, and uranyl acetate negative staining enables clear visualization of their characteristic cup-shaped or spherical appearance. However, to directly resolve the fine structure of the lipid bilayer, higher-resolution imaging techniques are typically required. Of note, cryo-electron microscopy better preserves the native state of vesicles and avoids deformation caused by drying processes; however, its widespread application is limited by the high cost of equipment and operational complexity [54].

Marker identification can be employed to confirm EV origin and assess purity [52,53]. Western blotting (WB) can be used to detect enrichment markers (such as CD9, CD63, CD81, TSG101, and Alix) and negative markers (such as calnexin and GM130) of extracellular vesicles. In purity assessment, non-EV-derived contaminants, including plasma proteins (e.g., albumin), lipoproteins, and protein aggregates, should also be fully considered. While conventional flow cytometry has inherent limitations in sensitivity for resolving small EVs, nano-flow cytometry has recently emerged as a powerful high-throughput tool capable of single-particle phenotyping and concentration determination [55,56]. Mass spectrometry (MS) can comprehensively profile EV protein composition and is suitable for discovering novel markers [57,58]. However, it requires expensive equipment, involves complex procedures, necessitates specialized expertise for data analysis, and typically cannot distinguish specific subtypes of small EVs (sEVs).

Particle size analysis primarily provides information on particle concentration and size distribution, morphological observation confirms the structural integrity of vesicles, and marker identification establishes their molecular identity and origin. Only through the combined characterization across these three dimensions can a reliable determination of the vesicular identity and purity of a sample be made.

### 2.3. MISEV Guidelines and Standardization Challenges

Having discussed individual characterization techniques, we now turn to the overarching guidelines that aim to standardize these measurements across laboratories.

To standardize research practices and enhance the comparability and reproducibility of data, the International Society for Extracellular Vesicles (ISEV) regularly publishes and updates the “Minimal Information for Studies of Extracellular Vesicles” (MISEV) guidelines, which have become the academic charter and methodological benchmark in this field [59]. The core recommendations of MISEV outline a framework for rigorous research and recommend the use of the generic term “extracellular vesicles” or clear labeling based on size differences as “small EV-enriched fractions” (<150 nm) and “large EV-enriched fractions” (>150 nm) [60]. They require detailed descriptions of experimental parameters for isolation and characterization to ensure process transparency. The guidelines suggest reporting the detection results of at least three positive markers and one negative marker to verify vesicle identity and purity [59]. Furthermore, they emphasize that functional experiments must be correlated with quantitative metrics of EVs, avoiding qualitative speculation, and strictly distinguish the boundaries between “EVs research” and “EVs-related research” to prevent conceptual overgeneralization [59]. Adherence to the MISEV guidelines is a fundamental reflection of academic rigor and a prerequisite for dialogue and reproducible validation across different studies.

However, the field still faces numerous standardization challenges [61]. Significant variations in isolation protocols across different laboratories lead to poor comparability of results. There is a lack of unified quality controls and reference materials. Subtype-specific markers remain unclear, making precise subtyping difficult [62]. Additionally, large-scale clinical studies create an urgent need for high-throughput, automated isolation technologies. These challenges constrain the translation of EV research towards clinical applications.

Beyond inter-laboratory variations, the inherent heterogeneity of EVs at the single-particle level poses a fundamental challenge to the batch-to-batch reproducibility of therapeutic outcomes [63,64]. Even within the same isolation batch, EVs can be divided into functionally distinct subpopulations that may exhibit markedly different in vivo biodistribution profiles and therapeutic activities [64]. Moreover, subtle fluctuations in parental cell culture conditions (e.g., passage number, cell confluency, oxygen tension, and serum batch differences) can lead to significant batch-to-batch alterations in the characteristics of these subpopulations, thereby contributing to inconsistent efficacy outcomes in preclinical models [65]. Consequently, current research efforts are increasingly focusing on single-particle EV characterization technologies and the development of reference standards tailored to specific subpopulations, with the aim of improving reproducibility [63].

## 3. Engineering Transformation Strategy for EVs

Natural EVs possess significant advantages, including low immunogenicity, high biocompatibility, and the ability to traverse biological barriers [66,67]. However, their clinical application still faces challenges. On one hand, targeting capability is insufficient; natural EVs tend to preferentially accumulate in the reticuloendothelial system, such as the liver and spleen, making it difficult for them to precisely reach diseased regions [68]. On the other hand, drug loading efficiency is relatively low, as EVs naturally encapsulate only small amounts of endogenous molecules during biogenesis, thereby limiting the loading capacity for exogenous therapeutic agents [69]. Additionally, the in vivo clearance rate is rapid, with natural EVs exhibiting a short circulation half-life, and they are quickly eliminated by phagocytic cells following a single administration, consequently compromising sustained therapeutic efficacy [70].

Therefore, engineering strategies for EVs primarily focus on three aspects: targeting capability, drug loading efficiency, and in vivo stability [71,72]. To enhance targeting, targeting ligands are introduced onto the EV surface, enabling specific recognition and binding to target cells or tissues [73]. Drug loading capacity is optimized through efficient loading of exogenous therapeutic agents, such as small-molecule compounds, nucleic acids, and proteins, either inside EVs or on their membrane surfaces [74]. To improve pharmacokinetics and reduce immune clearance, surface signals or molecules that prolong circulation half-life are modified to lower the rate of clearance by the immune system [75]. Based on the choice of modification nodes, existing strategies can be classified into three major categories: engineering parental cells before EV isolation, directly modifying isolated EVs, and constructing functionalized EVs via hybrid approaches. This chapter will elaborate on the principles, methods, and recent advances of the above engineering strategies.

### 3.1. Mother Cell Engineering

The core approach of parental cell engineering involves modifying donor cells. Prior to EV production, parental cells are subjected to genetic modification or drug preconditioning, enabling the subsequently secreted vesicles to naturally carry target molecules or acquire specific functionalities [76]. This strategy offers the advantage that the engineering events occur within the intracellular environment, thereby preserving the integrity of the vesicle membrane structure and the natural composition of the cargo to the greatest extent [77]. Moreover, once successfully established, this approach is amenable to large-scale, reproducible, and stable production [78].

Among the various parental cell engineering strategies, genetic modification to overexpress targeting proteins is the most widely used. Researchers fuse the gene sequence encoding a targeting ligand with that encoding an EV membrane scaffold protein. Following transfection into donor cells, the fusion protein is precisely loaded onto the EV membrane surface during the biogenesis process, achieving oriented display. Mahboube Shahrabi Farahani et al. genetically engineered human embryonic kidney 293T (HEK293T) cells to carry intercellular adhesion molecule 1 (ICAM-1) alongside lysosomal-associated membrane protein 2b (LAMP-2b) on their surface, thereby producing extracellular vesicles targeting CD3+ T cells [79]. The advantages of genetic modification include stable and reproducible display of targeting ligands, as the fusion protein is incorporated during EV biogenesis [77]. However, potential disadvantages include the risk of altering endogenous protein function, the need for stable transfection or viral vectors, and possible immunogenicity of the engineered surface proteins [80].

Drug pre-stimulation to modulate cargo represents another parental cell engineering approach. This strategy involves exposing donor cells to specific stimuli, such as drugs, hypoxia, or inflammatory factors, to induce responsive changes, leading to the secretion of EVs carrying corresponding functional molecules. Sanghee Shin et al. stimulated CD4+ T cells with IL-2, and the resulting secreted EVs more effectively activated the antitumor immune response of CD8+ T cells [81]. This strategy aimed to achieve targeted immunomodulation of T cells. Fei Wang et al. treated macrophages with IL-4 to induce their transformation into M2-type macrophages, thereby obtaining exosomes with anti-inflammatory factors [82]. Compared with genetic modification, this method is simpler in operation, obviating the need for complex vector construction and screening processes. However, the precision and batch-to-batch consistency of this approach are often constrained by fluctuations in stimulation conditions, making quantitative control difficult to achieve.

### 3.2. Post-Isolation Engineering of EVs

Direct modification after isolation refers to the functionalization of EVs by loading functional molecules into their interior or conjugating them onto their surface using physical, chemical, or biological methods following EV purification [66]. This strategy offers the advantage of flexible functionalization of specific EV batches without being limited by donor cell type, and it facilitates quality control and standardized operations.

With respect to functional molecule loading, research efforts have focused on how to efficiently introduce exogenous therapeutic agents into the interior of EVs. Currently, commonly employed methods include electroporation, co-incubation passive loading, sonication, and extrusion [83]. Electroporation utilizes a brief high-voltage electric field to create transient pores in the EV membrane, allowing nucleic acids or small-molecule drugs present in the surrounding solution to diffuse into the vesicles. This method exhibits high loading efficiency for nucleic acid-based drugs, but may induce EV aggregation or membrane damage [84,85]. The co-incubation method is suitable for hydrophobic small-molecule drugs. Simple mixing with EVs enables their embedding into the lipid bilayer via hydrophobic interactions, offering operational simplicity. However, the loading efficiency for hydrophilic drugs is relatively low [86]. Sonication and extrusion physically disrupt and then reseal the EV membrane, allowing drug entry into the vesicles. These methods achieve high loading efficiency, but may compromise the structural integrity of the vesicles [87]. Gwenola Tréton et al. conjugated the hydrophobic compounds curcumin and terbinafine with EVs through direct adsorption, achieving a loading capacity of up to 10^6^–10^7^ molecules per vesicle, thereby confirming the feasibility of the co-incubation method for loading hydrophobic molecules [88]. Lowe et al. pointed out that sonication exhibits the highest loading efficiency but significantly reduces particle yield, suggesting that the sonication process may lead to the destruction or loss of a portion of EVs [87]. Loading efficiency is typically quantified by encapsulation efficiency (EE%, defined as the percentage of the initial drug amount that is successfully loaded into EVs) and drug loading capacity (DLC, the amount of drug loaded per EV or per milligram of EV protein) [67]. In addition, the issue of cargo leakage cannot be overlooked. Small-molecule drugs loaded via passive incubation are prone to rapid release in serum; electroporation may induce nucleic acid aggregation; and sonication can lead to sustained leakage due to membrane damage. Different loading methods exert significantly distinct effects on EV membrane integrity and functionality; therefore, in practical applications, optimization should be performed based on drug properties and subsequent intended uses [89].

**Figure 3:**
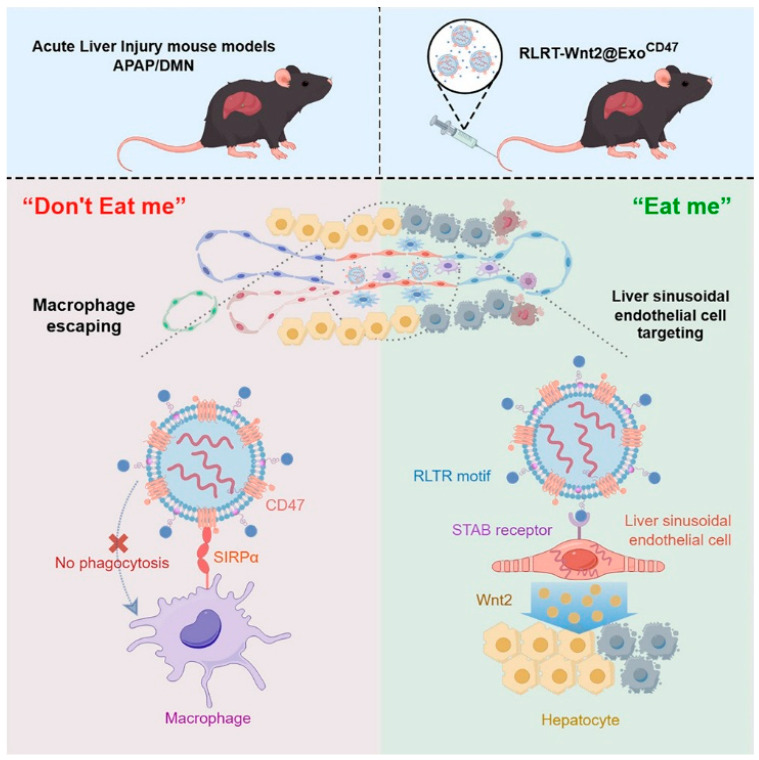
Schematic illustration of the “Don’t Eat Me/Eat Me” dual-targeting strategy of engineered exosomes for acute liver injury treatment [96]. Engineered EVs modified with surface RLTR peptide (RLTR-Wnt2@ExoCD47) can achieve targeted recognition of liver sinusoidal endothelial cells (LSECs) and efficiently deliver Wnt2 mRNA, while simultaneously evading phagocytic clearance by macrophages via the CD47 molecules displayed on their surface. Specifically, the CD47 molecules on the EV surface specifically bind to signal regulatory protein α (SIRPα) receptors on macrophages, transmitting the classic “Don’t eat me” inhibitory phagocytic signal, thereby significantly reducing the risk of non-specific clearance of the engineered exosomes by the immune system in vivo.

Surface targeting modification techniques aim to confer precise recognition of diseased sites by conjugating targeting ligands onto the EV membrane surface [90]. The main methods include chemical conjugation, lipid insertion, and metabolic labeling [91]. Chemical conjugation employs reactions such as click chemistry or amidation to covalently link targeting ligands to EV membrane proteins or lipids. This approach is operationally flexible and broadly applicable, but may exert certain effects on EV surface properties [92]. Lipid insertion involves coupling targeting ligands with hydrophobic anchor molecules, followed by insertion into the EV lipid bilayer via hydrophobic interactions [93,94]. This method is mild in operation and better preserves membrane protein functionality. Metabolic labeling involves adding azide-containing sugar or lipid precursors to the culture medium, allowing their metabolic incorporation into the membrane components of secreted EVs [95]. Lin Wang et al. employed a genetic engineering strategy to precisely regulate the expression density of CD47 on the exosomes’ surface, and its overexpression effectively inhibited macrophage phagocytosis and significantly prolonged the liver retention half-life of engineered exosomes, providing a precise paradigm for improving exosomes’ pharmacokinetic performance, as shown in Figure 3 [96]. Maria Chiara Ciferri et al. developed a standardized method for functionalizing EV membranes via copper-free click chemistry, successfully achieving fluorescent labeling of plasma-derived EVs [97]. Rimsha Bhatta et al. established a simple and versatile metabolic labeling technique that installs unique chemical tags onto EVs [98]. This metabolic tagging technology provides a universal platform for generating chemically tagged EVs, modulating EVs–cell interactions, and developing potent EVs-based cancer vaccines [98]. These surface chemical tags enable conjugation with molecules via efficient click chemistry, facilitating EV tracing and targeted regulation. Chemical conjugation offers flexibility but may affect EV surface properties and membrane fluidity [99]. Lipid insertion is mild and preserves protein conformation but relies on hydrophobic interactions that may be less stable in vivo [100]. Metabolic labeling provides stable covalent attachment but requires pre-labeling of donor cells and is more complex. Compared to cargo loading methods (electroporation, sonication), surface modification strategies generally have minimal impact on EV integrity, though optimization is needed to avoid excessive modification that could alter EV targeting or clearance [101].

Hui Yang et al. developed a universal micro-nanofluidic platform termed ExoSE, which decouples the EV surface engineering process into two independent modules: “loading” and “mixing” [102]. The loading module utilizes a high-throughput parallel nanofluidic structure to achieve efficient lipid insertion into EVs via mechanical transient perforation, attaining a lipid insertion efficiency exceeding 97%, which is markedly superior to traditional co-incubation methods [102]. The mixing module facilitates rapid conjugation between ligand molecules and membrane-inserted lipids, thereby enabling efficient targeting modification [102]. This technique enables subsequent conjugation with fluorescent dyes or targeting ligands via click chemistry for EV tracing and targeted delivery.

### 3.3. Construction of Hybrid EVs

Hybrid EVs represent a strategy that fuses EVs with synthetic nanomaterials or vesicles derived from other sources to construct hybrid nanoparticles possessing both natural characteristics and artificial functionalities [103,104]. The core of this strategy lies in breaking through the functional limitations of native EVs, enabling flexible assembly and synergistic cooperation of multiple functional modules [105].

Liposome-EV hybrid vesicles are a relatively well-studied class of hybrid systems. Their preparation typically involves co-incubating EVs with synthetic liposomes, followed by physical methods such as freeze–thaw cycles or extrusion to promote membrane fusion between the two, thereby forming structurally stable hybrid vesicles [106]. These hybrid systems not only retain the natural membrane proteins and targeting capability of EVs but also incorporate the advantages of liposomes, including high drug loading efficiency and controlled release, achieving enhanced drug loading capacity and improved drug release profiles. So Yun Kim et al. prepared nanoscale uniform hybrid vesicles, incorporated anticancer drugs, and evaluated their physicochemical properties and cellular internalization in breast cancer cells [107]. Jong-Soo Choi et al. isolated EVs from adipose-derived stem cells (ADSCs) and fabricated hybrid EVs by fusing them with cholesterol-doxorubicin liposomes via the freeze–thaw method [108].

Despite their potential, hybrid EVs face challenges such as batch-to-batch variability in fusion efficiency, potential loss of native EV surface proteins during membrane mixing, and the need for rigorous characterization to confirm true fusion rather than simple co-aggregation. Additionally, scaling up hybrid EV production remains technically challenging.

Hybrid EVs integrate natural and synthetic components into a unified entity via membrane fusion. In contrast, the recently emerged biomimetic engineering strategy adopts a different design concept, which employs natural cell membranes or extracellular vesicle membranes for surface coating modification of synthetic nanoparticle cores.

### 3.4. Biomimetic Engineering Strategy

In addition to the aforementioned mainstream engineering strategies, a nanodelivery strategy centered on the design concept of “biomimetic camouflage” has attracted widespread attention. This strategy employs natural cell membranes or cell membrane-derived vesicles to coat the surface of nanoparticles, thereby endowing the nanocarriers with the inherent surface biological properties of the source cells, aiming to achieve immune evasion and precise targeted delivery [109,110,111]. Such biomimetic nanocarriers typically consist of a synthetic nanoparticle core coated with animal-derived cell membrane components, effectively mimicking the biological behavior of natural cells in vivo. Compared with natural EVs, biomimetic nanoparticles achieve large-scale production through chemical synthesis, fundamentally addressing bottlenecks such as low yield and complicated purification, while simultaneously prolonging in vivo circulation time and achieving targeted accumulation at diseased sites by virtue of the source cell membrane. However, the extent of immune evasion and targeting precision may vary depending on membrane source and coating integrity [112]. Challenges remain in batch-to-batch consistency, long-term stability, and potential immunogenicity of xenogeneic membrane components [113].

The construction of cell membrane-coated biomimetic nanoparticles generally employs membrane extrusion or sonication fusion methods, in which pre-isolated and purified cell membrane fragments are physically wrapped around the synthetic nanoparticle core to form a single-layer or multi-layer membrane-coated structure [114]. By integrating natural cell membrane components with synthetic nanomaterials, a biomimetic nanodelivery platform that possesses both the surface characteristics of natural cells and the controllable advantages of artificial materials can be rapidly obtained [115,116,117]. In addition, some researchers have directly prepared extracellular vesicle mimetics from intact cells using technical approaches such as mechanical extrusion. Qihong Cheng et al. co-extruded cell membranes with doxorubicin (DOX) via physical extrusion to prepare DOX-loaded EV mimetics (CAR-EVMs@DOX) [118]. Through the non-invasive intranasal route, these mimetics efficiently targeted and inhibited glioblastoma (GBM) growth while cleverly circumventing the blood–brain barrier (BBB) and the systemic toxicity of chemotherapeutic drugs, offering a novel strategy with high clinical translation potential for GBM treatment [118].

**Figure 4:**
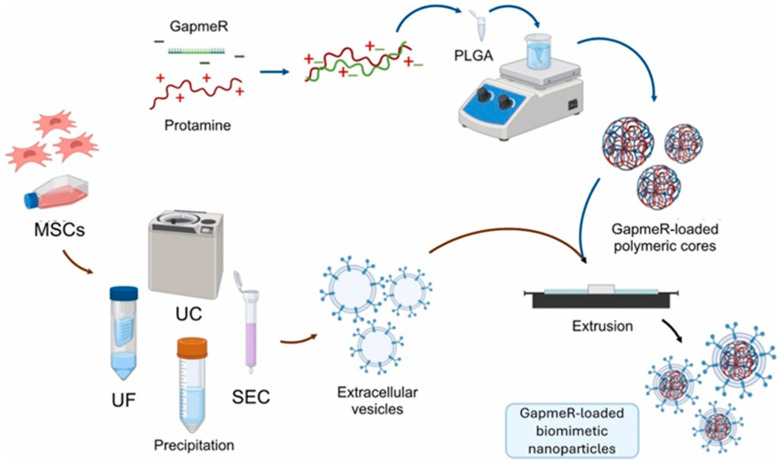
Schematic view of the elaboration process of antisense oligonucleotide (GapmeR)-loaded, mesenchymal stem cells (MSCs) EV-membranecoated biomimetic nanoparticles (BMNPs-G) [109]. EVs were isolated from MSCs by UC, UF, SEC, or precipitation methods. Meanwhile, GapmeR was condensed with protamine and encapsulated into a polylactic-co-glycolic acid (PLGA) polymer core. Finally, the drug-loaded polymer core was co-extruded with the aforementioned EVs to form core–shell structured biomimetic nanoparticles for gene therapy.

Natural EVs themselves also constitute an indispensable component of the cell membrane coating biomimetic strategy [119]. Researchers often utilize the EV membrane structure as a “camouflage outer layer” to coat synthetic nanoparticles or other therapeutic agents [120]. This EV membrane coating-based functionalization strategy combines the excellent biocompatibility of natural EVs with the high drug-loading efficiency of synthetic nanoparticles, representing a cutting-edge area where biomimetic engineering and EV research deeply intersect. Jan Van Deun et al. demonstrated that EV-membrane-coated gold nanoparticles effectively reduced macrophage uptake while simultaneously enhancing autologous uptake efficiency [121]. Erik Briffault et al. designed and constructed a simple and highly tunable biomimetic drug delivery nanosystem comprising a polymeric nanoparticle core encapsulated by an extracellular vesicle-derived membrane, as shown in Figure 4 [109]. However, the EV membrane coating approach has certain limitations. Batch-to-batch consistency of the coated cell membranes cannot yet be fully guaranteed, large-scale production processes meeting clinical-grade application requirements have not yet been well established, and the stability of the EV membrane structure itself as well as its long-term in vivo safety remain to be systematically and thoroughly evaluated.

From a stringent biomimetic engineering perspective, the transition from the direct utilization of intact extracellular vesicles to synthetic core–shell cell membrane-camouflaged nanoparticles represents a major technological leap, yet simultaneously highlights several critical research gaps [122]. Although physical extrusion and sonication techniques can successfully coat biological membranes onto abiotic core surfaces, they often struggle to maintain the correct native exofacial topological orientation of transmembrane proteins, which is essential for precise cellular recognition [112]. The random orientation of membrane fragments remains a core limiting factor that constrains the theoretical targeting advantages of biomimetic systems. Moreover, current research frameworks lack a standardized set of metrics for quantitatively assessing coating efficiency and shell structural integrity at single-particle resolution. Future research urgently needs to shift from empirical formulation screening to a mechanistic understanding of the interfacial thermodynamics governing membrane–nanoparticle interactions, thereby enabling predictable and highly reproducible biomimetic delivery.

### 3.5. Functional Evaluation of Engineered EVs

The evaluation of engineered EVs should be systematically assessed from multiple dimensions. Regarding targeting capability, confocal microscopy and flow cytometry are typically employed at the cellular level to examine the specific binding efficiency of engineered EVs to target cells [123,124]. Subsequently, in vivo imaging techniques are utilized to track their tissue distribution and targeted accumulation at diseased sites in animal models [125]. Zhen Sun et al. constructed aminoethyl anisamide-modified EVs that specifically recognized and efficiently bound to activated hepatic stellate cells, and cellular assays demonstrated that the binding efficiency of these modified EVs was significantly higher than that of the unmodified group [126].

For drug loading efficiency, the core metric is the amount of encapsulated drug molecules per unit number of EVs. Quantitative techniques such as high-performance liquid chromatography (HPLC) or mass spectrometry are commonly employed for detection [127,128]. Additionally, the potential impact of the loading process on EV membrane integrity and the bioactivity of the encapsulated drugs should be assessed. Membrane integrity can be evaluated using fluorescent dyes or by comparing EV size distribution before and after loading via NTA or cryo-TEM. Drug bioactivity can be assessed by in vitro release assays (e.g., dialysis or SEC) followed by functional assays [87]. In vivo pharmacokinetic studies typically utilize fluorescence labeling or radioisotope labeling to monitor the clearance kinetics and tissue distribution profiles of engineered EVs in the bloodstream [129,130]. Cheng-Hsiu Lu et al. developed a simple, stable, and promising radiolabeling probe, 111In-oxine, for dynamic in vivo monitoring of exogenously administered Wharton’s Jelly Mesenchymal Stem Cell-derived Extracellular Vesicles (MSC-EVs) [131].

The safety evaluation of engineered EVs represents a critical consideration for their clinical translation, with particular attention paid to the potential for increased immunogenicity or toxicological risks introduced by engineering strategies [132]. Animal studies are instrumental in assessing the in vivo safety profile of these modified EVs, thereby demonstrating their potential for therapeutic applications [133]. Animal study results indicated that engineered EVs modified using the ExoSE platform did not induce significant liver or kidney dysfunction, preliminarily confirming their favorable in vivo safety profile [102]. As EV engineering strategies continue to advance, the development of systematic evaluation frameworks is essential to accelerate their progression toward clinical use.

Although various engineering strategies each possess unique advantages, direct horizontal comparisons among them are often complicated by differences in EV sources, types of cargo loaded, and detection conditions [134]. Importantly, most studies report positive outcomes, which may lead to publication bias [135]. Unresolved issues include the long-term stability of surface modifications in vivo, the potential immunogenicity of introduced synthetic components, and batch-to-batch consistency of engineered EVs derived from genetically modified parental cells.

## 4. Applications of Engineered EVs in Disease Therapy

In recent years, the therapeutic potential of engineered EVs has been evaluated across a variety of disease models, with research primarily focusing on three major directions: oncology, central nervous system disorders, and tissue repair. Extensive preclinical explorations have been conducted, and some research findings have advanced to clinical trial stages. This section highlights the state-of-the-art applications in oncology, neurology, and regenerative medicine, and emphasizes the rationale behind engineering choices for each scenario.

### 4.1. Engineered EVs in Tumor Treatment

Engineered EVs have emerged as a research hotspot in the field of cancer therapy. Although naturally derived EVs possess certain tumor-homing properties, their targeting efficiency is relatively low, and their capacity to penetrate the immunosuppressive tumor microenvironment remains limited [136]. Through engineering strategies such as surface modification and cargo engineering, multifunctional EV delivery platforms integrating targeted recognition, drug loading, and immunomodulation have been successfully constructed [137].

In the context of chemotherapeutic drug delivery, engineered EVs exhibit significant advantages, enabling efficient loading of various chemotherapeutic agents, including doxorubicin (DOX) and paclitaxel [138]. Following surface modification with specific ligands, these EVs achieve enhanced accumulation at tumor sites, substantially increase local drug concentrations, and effectively reduce systemic toxicities [139]. Tao Sun et al. developed a bacterial outer membrane vesicle (OMV)-based nanodelivery platform modified with Angiopep-2 peptide, which successfully crossed the blood–brain barrier to precisely deliver DOX to glioblastoma lesions [140]. This platform was further co-loaded with CD47-siRNA, and by silencing CD47 gene expression to enhance macrophage phagocytic activity, it achieved synergistic enhancement of chemotherapeutic efficacy and immune response [140].

In the field of nucleic acid drug delivery, the application of engineered EVs is progressively overcoming the clinical translation bottlenecks of RNA interference (RNAi) therapy [141]. As natural nucleic acid carriers, EVs effectively protect small interfering RNA (siRNA) from nuclease degradation and facilitate its efficient intracellular transport. Jun Yang et al. designed an engineered exosome system modified with acid-cleavable transferrin for co-delivery of transforming growth factor-β (TGF-β) siRNA and DOX [142]. This delivery system specifically recognized transferrin receptors on the surface of blood–brain barrier endothelial cells, underwent responsive detachment in the acidic microenvironment to achieve transcytosis, and further targeted glioblastoma cells upon entering the brain parenchyma [142]. Animal study results demonstrated that this strategy significantly downregulated TGF-β expression, effectively remodeled the immunosuppressive microenvironment, and synergistically enhanced the chemotherapeutic efficacy of doxorubicin as well as the antitumor immune response [142].

With the continuous deepening of immunotherapy concepts, research on engineered EVs is expanding toward combination immunotherapy [143,144]. The aforementioned bacterial outer membrane vesicle platform developed by Tao Sun et al., through the triple synergistic mechanism involving natural immune adjuvants carried by the vesicles themselves, co-loaded CD47-siRNA, and DOX, collectively overcame the tumor immune resistance barrier and significantly improved the overall efficacy of immunotherapy [140]. This study provides a novel strategic reference and theoretical basis for the application of engineered EVs in the immunotherapy of refractory tumors.

Despite the encouraging progress made in the aforementioned studies, certain inconsistent experimental findings also warrant attention. Some engineered EV formulations that exhibited significant antitumor activity in subcutaneous xenograft models failed to reproduce comparable therapeutic efficacy in orthotopic or patient-derived xenograft models [145]. This discrepancy highlights the substantial constraint imposed by the tumor microenvironment barrier on the intratumoral penetration efficiency and biological function of EVs [146]. Furthermore, certain EV-based therapies designed to suppress immune responses unexpectedly triggered immune activation rather than effectively exerting immunosuppressive regulatory effects in animal models with specific genetic backgrounds [147]. These observations suggest that personalized therapeutic evaluation based on individual genetic backgrounds and tumor immune phenotype heterogeneity holds important guiding significance for the precise clinical application of engineered EVs.

### 4.2. Engineered EVs for the Treatment of Central Nervous System Diseases

The treatment of central nervous system (CNS) diseases has long been constrained by the BBB, a highly selective physiological structural barrier. It is important to clarify that while certain naturally occurring EVs possess intrinsic BBB-crossing ability, this capacity is often limited and highly heterogeneous across different EV subpopulations [148]. Therefore, how to further enhance the targeted delivery capability of EVs across the BBB through engineering modification strategies has become one of the current research hotspots in this field [149,150].

Regarding strategies for crossing the blood–brain barrier, researchers have established multiple targeted modification approaches. In addition to the aforementioned transferrin receptor targeting strategy, peptide fragments derived from the rabies virus glycoprotein (RVG) are also widely utilized to enhance the brain-targeting capability of EVs [151]. The RVG peptide specifically recognizes and binds to the nicotinic acetylcholine receptor (nAChR), and through receptor-mediated transcytosis, guides engineered EVs to efficiently traverse the blood–brain barrier and enter the brain parenchyma [152]. Yazhou Wang et al., by constructing a hybrid cell system of mesenchymal stem cells and neutrophils, achieved large-scale production of engineered exosomes possessing both inflammatory targeting chemotactic properties and tissue repair functions [153]. These hybrid cell-derived exosomes inherited the rapid responsiveness of neutrophils to inflammatory signals, enabling them to precisely cross the blood–brain barrier and achieve effective accumulation in brain lesion regions in APP/PS1 double-transgenic Alzheimer’s disease (AD) model mice [153].

Engineered EVs exhibit significant application potential in the interventional treatment of neurodegenerative diseases. The core pathological features of Alzheimer’s disease (AD) are primarily characterized by abnormal deposition of amyloid-β (Aβ), chronic neuroinflammation, and progressive neuronal injury [154]. Leelavathi N Madhu et al. demonstrated that EVs secreted by induced pluripotent stem cell-derived neural stem cells (NSCs) could significantly alleviate the pro-inflammatory cascade response mediated by AD pathology-associated microglia [155]. While these are natural EVs rather than engineered ones, their therapeutic effects identify key functional properties that can be enhanced by engineering strategies. Furthermore, Chen Jin et al. utilized stem cell-derived exosomes to encapsulate upconversion nanoparticles (UCNPs) in combination with specific targeting molecules, successfully achieving targeted clearance of Aβ aggregates [156]. The results indicated that this strategy markedly reduced the cytotoxicity induced by misfolded Aβ aggregates in neuronal cells [156].

### 4.3. Applications of Engineered EVs in Tissue Repair and Regenerative Medicine

Engineered EVs have seen particularly rapid progress in clinical translation research within the field of tissue repair. MSC-derived EVs inherently possess certain tissue-regenerative potential, and engineering strategies can further amplify their therapeutic efficacy while substantially broadening their application scenarios [157,158].

In the area of wound healing and skin regeneration, engineered EV-based formulations have advanced to clinical research stages. Jodi P. Gurney et al. developed engineered adipose-derived stem cell (ADSC)-derived EVs produced at scale using a bioreactor system [159]. These engineered EVs exhibit a unique bioactivity profile, effectively reducing the release of inflammatory cytokines, stimulating collagen synthesis, and activating the proliferation and migration of keratinocytes and fibroblasts [159]. In a mouse skin excisional wound model, topical application of this formulation significantly promoted wound closure, enhanced epidermal keratinization, and increased collagen deposition density, thereby comprehensively improving wound healing rates. In a clinical case study of a chronic diabetic foot ulcer, local treatment with engineered EVs resulted in complete wound closure and effective dermal regeneration, demonstrating promising clinical therapeutic potential [159].

**Figure 5:**
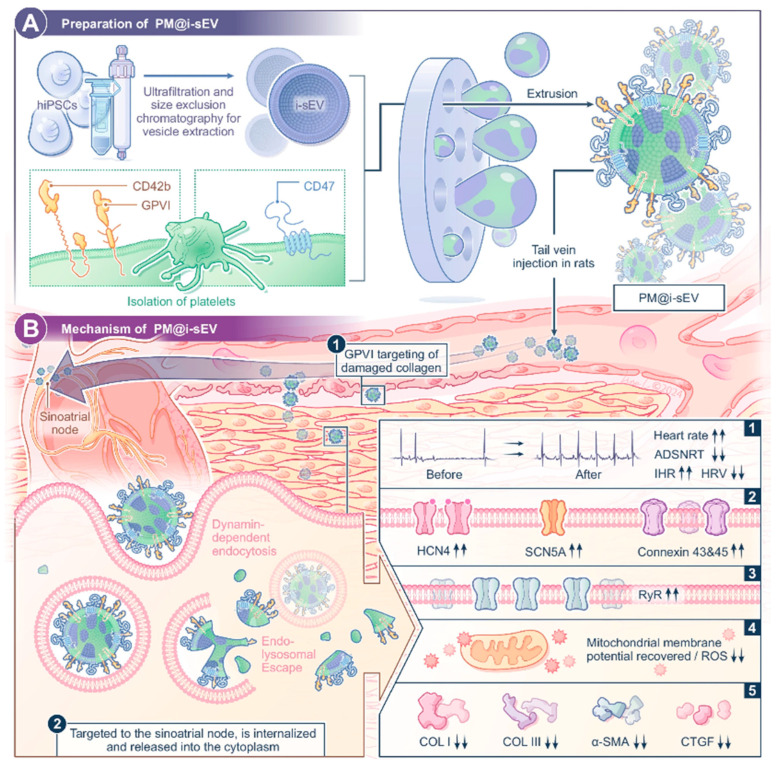
(**A**) hiPSC-sEVs were isolated by ultrafiltration and size-exclusion chromatography, and platelet membrane vesicles (with CD42b, GPVI, CD47) were prepared by freeze–thaw cycles. The components were then fused into PM@i-sEV via extrusion. (**B**). Intravenously administered PM@i-sEV target the ischemic sinoatrial node via GPVI–collagen binding; after cellular uptake and endosomal escape, they restore sinoatrial node function, enhance mitochondrial activity, and reduce fibrosis [160].

Cardiovascular tissue repair represents another important research direction for engineered EV applications. Hanfeng Liu et al. successfully constructed a delivery system of small EVs derived from human induced pluripotent stem cells (iPSCs) fused with platelet membranes [160]. As shown in Figure 5, this engineering strategy effectively endowed iPS-EVs with the inherent collagen-targeting binding capability and immune evasion properties of platelet membranes [160]. In a rat model of sinus node dysfunction, the accumulation of modified EVs in the sinus node region was 3.1-fold higher than that of the unmodified group, local fibrosis was significantly reduced, and both heart rate and intrinsic pacemaker function were markedly restored [160]. The study demonstrated that these engineered EVs could effectively inhibit aberrant fibroblast activation and protect cardiomyocytes from oxidative stress-induced damage [160].

In the context of liver and bone tissue repair, engineered EVs also exhibit broad application prospects. Lingyan Yang et al. constructed engineered sEVs co-displaying both WNT3A and RSPO1 ligands on their surface [161]. This engineering strategy synergistically activated the hepatic WNT/β-catenin signaling pathway [161]. In models of acute liver injury, chronic liver fibrosis, and age-related liver regeneration impairment, these sEVs effectively promoted hepatocyte proliferation, alleviated tissue damage, and accelerated the restoration of liver function, demonstrating significant potential for liver regeneration therapy [161]. Junhao Sui et al. conjugated regulatory T cell (Treg)-derived exosomes with bone morphogenetic protein-2 (BMP-2) mimetic peptides via click chemistry, successfully constructing a nanotherapeutic platform possessing dual functions of immunomodulation and osteogenic induction, and validated its superior repair efficacy in a rat bone defect model [162]. This strategy not only effectively circumvented complications associated with conventional BMP-2 therapy, such as inflammatory responses and heterotopic ossification, but also provided a new technical paradigm for immuno-osteogenic synergistic therapy [162]. However, the therapeutic efficacy of this platform has currently only been validated in animal models. Future studies are warranted to further evaluate its therapeutic effects in complex pathological microenvironments such as diabetes and osteoporosis, and to optimize large-scale preparation processes to facilitate its clinical translation [162].

### 4.4. Summary

In summary, engineered EVs exhibit broad application prospects in three major research fields: tumor therapy, central nervous system disease intervention, and tissue repair. In response to the therapeutic needs of different disease types, engineering design strategies have followed distinct development trajectories. Tumor therapy emphasizes the synergistic effect of precise targeting and immunomodulation, whereas central nervous system diseases focus on the integration of blood–brain barrier crossing capability and neuroprotective functions. Tissue repair, on the other hand, prioritizes the enhancement of regenerative potential and the balance of immune microenvironment regulation. The above research progress not only validates the therapeutic potential of engineered EVs but also accumulates an important foundation for subsequent clinical translation.

## 5. Challenges in Clinical Translation

### 5.1. Large-Scale Production and Quality Control of Engineered EVs

The clinical translation of engineered EVs first faces the severe challenge of large-scale production. The yield of naturally secreted EVs is extremely limited, and the quantities provided by traditional cell culture systems fall far short of meeting the requirements for clinical therapeutic doses. The EV dose required for a single treatment reaches a remarkably high order of magnitude, and the specific dose varies significantly depending on the indication, route of administration, and cell source [163,164]. The application of automated bioreactor systems provides a critical avenue to overcome this production bottleneck. By enabling precise regulation of culture microenvironment parameters, these systems allow cells to continuously and efficiently secrete EVs under three-dimensional culture conditions that more closely mimic the physiological state, thereby achieving a significant leap in volumetric yield. Currently, combined strategies employing three-dimensional cell culture technologies, microcarrier-based suspension culture systems, and continuous perfusion bioreactors have been demonstrated to substantially enhance cell culture density and volumetric EV production, laying the technological foundation for the industrial-scale manufacturing of EVs for clinical applications [165,166,167].

Isolation and purification processes represent another bottleneck in large-scale production. Ultracentrifugation, when scaled up for industrial manufacturing, not only suffers from limited throughput but also faces inherent limitations such as poor linear scalability, a tendency to damage vesicle structures, and a nonlinear decrease in yield as the volume increases [168]. In addition to ultracentrifugation, several emerging and scalable purification methods are gaining increasing attention. Tangential flow filtration (TFF) enables continuous processing of large sample volumes under very low shear stress, making it suitable for industrial-scale production [169]. SEC offers good scalability and preserves the integrity of EVs, though its throughput remains relatively limited [169]. Affinity-based methods can provide high purity, but are costly and currently have low throughput. Microfluidic platforms, while highly efficient at the laboratory scale, still face challenges when scaled up to clinical volumes [170]. An ideal process should achieve high recovery rates while effectively removing impurities such as lipoproteins and protein aggregates, and maintaining the structural integrity and biological activity of EVs.

Standardization of quality control is a core prerequisite for clinical translation [171]. Given the heterogeneous nature of EVs, establishing a unified quality control framework is particularly challenging. International guidelines, such as MISEV 2023, have provided the academic community with the most authoritative reference framework for EV characterization [59]. A comprehensive quality control system should encompass multiple dimensions, including starting materials, critical process parameters, EV characterization, purity assessment, biological activity, and stability studies. Furthermore, the heterogeneity of EVs must be recognized as a critical quality attribute. Two batches with identical total particle numbers may contain vastly different proportions of active subpopulations, leading to divergent therapeutic outcomes. Therefore, quality control frameworks must move beyond bulk measurements and incorporate subpopulation analysis to ensure batch-to-batch consistency [172,173].

### 5.2. Safety Assessment of Engineered EVs

The safety evaluation of engineered EVs represents the fundamental prerequisite determining their eligibility for clinical translation. Compared with synthetic nanoparticles, although EVs inherently possess relatively low immunogenicity and favorable biocompatibility, as allogeneic biological products, their potential safety risks still require systematic assessment [171].

#### 5.2.1. Immunogenicity and Toxicological Responses

Immunogenicity and toxicological responses are the primary factors to be considered [174]. Engineering processes may introduce novel immunogenic epitopes; therefore, it is necessary to systematically evaluate the impact of engineered EVs on immune cell subset distribution, the risk of cytokine release, and potential toxicological reactions in preclinical studies. Seoyeon Hyun et al. conducted a safety assessment of a single intravenous infusion of escalating doses of engineered EVs (ILB-202, derived from HEK293 cells and loaded with the IκBα super-repressor) in 18 healthy volunteers [175]. The results demonstrated favorable in vivo safety of ILB-202, with no severe adverse events or dose-limiting toxicity observed [175]. This finding underscores the unique application advantage of engineered EVs in achieving precise immunomodulation.

The in vivo biodistribution profile of EVs also warrants particular attention. Following systemic administration, EVs predominantly accumulate in the liver tissue, so hepatotoxicity evaluation should be a routine component of the safety assessment framework. Jing Zhang et al. systematically compared the in vivo metabolic fate of natural EVs, EVs with ITGB1 alone knocked out, and EVs subjected to combined modification by knocking out integrin β1 (ITGB1), a key adhesion molecule on the EV surface, in conjunction with deglycosylation treatment [176]. The results demonstrated that engineering modifications significantly reduced the accumulation level of EVs in liver tissues. Notably, ITGB1 knockout alone did not substantially improve the in vivo circulation time; however, EVs subjected to combined ITGB1 knockout and deglycosylation (ITGB1^−^Deg EVs) exhibited superior systemic exposure accompanied by a marked reduction in liver accumulation [176]. Amer F. Saleh et al. reported that even at exposure concentrations as high as 5 × 10^10^ EVs/mL, no significant hepatotoxicity was observed, and both liver function-related biochemical indices and histopathological examination of liver tissues revealed no notable abnormalities [177].

Equally importantly, although it has been widely accepted that EVs possess extremely low immunogenicity, several recent studies have observed dose-dependent cytokine (e.g., IL-6, TNF-α) release responses following intravenous administration of certain engineered EVs, particularly those derived from allogeneic cell lines or extensively modified with synthetic ligands [178]. These experimental findings suggest that low immunogenicity is not an inherent property shared by all EVs; rather, the risk of immunogenicity is highly dependent on the integrated selection of cell source, engineering modification strategy, and final product purification method.

#### 5.2.2. Potential Tumorigenic Risk

Tumorigenic risk represents another safety concern that cannot be overlooked. For EV products derived from tumor cell lines or genetically modified immortalized cells, legitimate safety concerns inevitably arise within the research community regarding their potential tumorigenicity or tumor-promoting capacity [179]. Prior to clinical application, it is imperative to confirm whether the final engineered EV product contains residual oncogenic nucleic acid sequences or carries pro-proliferative signals, thereby effectively excluding potential tumorigenic risks. Fei Wang et al. demonstrated that nanovesicles derived from A549 lung cancer cells exhibited a propensity to promote tumorigenesis and metastasis, whereas nanovesicles derived from umbilical cord mesenchymal stem cells (UCMSCs) did not display such tumorigenic characteristics [180].

### 5.3. Regulatory Policy and Landscape for Engineered EVs

Regulatory science for EV-based therapeutics has emerged as a cutting-edge international frontier owing to the inherent heterogeneity and compositional complexity of these products. Currently, major pharmaceutical regulatory authorities worldwide have yet to issue specific technical guidance dedicated to EV-based therapeutics, and this regulatory gap has, to some extent, constrained their clinical development [181].

In China, the specific technical evaluation guidelines dedicated to EV-based therapeutics are still under development. Gangling Xu et al., drawing on their experience in quality control of biotechnological drugs, have proposed a quality control strategy and general principles for the nonclinical evaluation of EV-based therapeutics, actively advancing relevant standardization research [171].

Internationally, the ISEV continues to update the MISEV guidelines, with the latest version, MISEV2023, providing academic consensus on the nomenclature, isolation, characterization, and standardization of EV studies [59]. Concurrently, ISEV has established a Regulatory Affairs Task Force specifically dedicated to facilitating the clinical translation and regulatory alignment of EV-based therapeutic strategies. Nevertheless, a gap persists between academic guidelines and regulatory requirements, as the former emphasize research recommendations while the latter establish market access standards [182]. The U.S. Food and Drug Administration (FDA) has accepted multiple investigational new drug (IND) applications related to EVs and is accumulating regulatory experience through case-by-case reviews [181]. However, as of now, no EV-based therapy has yet received FDA approval [181].

### 5.4. Summary

As a next-generation natural nanoscale drug delivery platform, engineered EVs are accelerating their transition from the laboratory toward clinical translation. Continuous advancements in scalable production, quality control, safety assessment, and regulatory policies are laying a solid foundation for this transition. Although challenges remain, ongoing technological breakthroughs and the progressive refinement of regulatory science position engineered EVs to offer innovative therapeutic solutions for oncology, neurological disorders, and tissue injury repair, ultimately benefiting a broader patient population.

## 6. Conclusions

EVs, as natural nanomaterials, have undergone a paradigm shift in perception from “cellular waste” to “intercellular communication messengers” and subsequently to “drug delivery platforms”. This evolution has not only deepened our understanding of fundamental biological processes but also opened up novel avenues for disease therapy. This review primarily summarizes the research progress of engineered EVs in disease treatment, covering four aspects: isolation and characterization, engineering strategies, preclinical applications, and the challenges of clinical translation.

Engineering strategies represent the core approach to overcoming the clinical application bottlenecks of natural EVs. Through parental cell genetic modification, direct post-isolation modification, and the construction of hybrid EVs, researchers have endowed EVs with precise targeting capabilities, efficient drug loading characteristics, and improved pharmacokinetic profiles. These modifications enable EVs to cross the blood–brain barrier for drug delivery, precisely recognize tumor cells, synergistically activate antitumor immunity, and exert regenerative effects in tissue repair. Preclinical studies have achieved promising results in tumors, central nervous system diseases, and tissue regeneration, with some engineered EV formulations having entered clinical trials.

Nevertheless, to successfully translate EVs into approved clinical therapeutic products, the field urgently needs to move beyond empirical optimization and address the following five key research gaps and technological bottlenecks:

1. Heterogeneity control and subpopulation resolution: current isolation methods yield EV populations with a high degree of heterogeneity. The lack of high-resolution molecular markers capable of specifically distinguishing distinct EV subpopulations significantly obscures the correlation between vesicle molecular phenotypes and therapeutic outcomes, thereby posing a severe challenge to achieving batch-to-batch standardization at both scientific and regulatory levels.
2. Scalable manufacturing process intensification and process analytical technology (PAT): the transition from static bench-top cultures to three-dimensional continuous perfusion bioreactor systems, while greatly increasing volumetric yield, simultaneously introduces significant shear stress fluctuations that may alter the molecular characteristics of secreted EVs. Deploying real-time PAT for in-line monitoring of critical quality attributes of EVs during continuous harvesting remains an unmet core need in current industrial production.
3. Standardization of characterization and quality control: although the MISEV 2023 guidelines have systematically standardized reporting requirements, the international regulatory environment still lacks common reference materials. This absence severely hinders the direct calibration and data comparison across laboratories of advanced single-particle characterization tools.
4. High-resolution in vivo fate tracing: conventional macroscopic imaging modalities (e.g., optical tracing or radionuclide labeling) cannot capture the real-time dynamic process of EV cargo release at the single-cell level. There is an urgent need to develop deep-tissue imaging platforms capable of non-invasively resolving intracellular endosomal escape versus lysosomal degradation, which is critical for mechanistically confirming the therapeutic action of EVs.
5. Differentiated regulatory framework construction: currently, regulatory agencies predominantly evaluate engineered EVs using mismatched frameworks adapted from guidelines for cell therapy products or synthetic liposomes. Establishing a dedicated regulatory science system that fully addresses the unique biological complexity of engineered and biomimetic hybrid vesicles constitutes the final critical barrier to successfully crossing the clinical translation chasm.

Looking forward, with a deeper understanding of EV heterogeneity, the emergence of intelligent engineering strategies, the cross-integration of multidisciplinary technologies, and the continuous advancement of international standardization, engineered EVs are expected to evolve into a mature precision therapy platform. It is anticipated that in the near future, these naturally derived nanocarriers will truly benefit patients, playing their unique value in the treatment of tumors, neurodegenerative diseases, and tissue injury repair.

## Figures and Tables

**Table 1:** Comparison of Common EV Isolation Methods.

Method	Time	Ease of Operation	Sample Volume Capacity	EVs Bioactivity	Purity
UC	Long	Difficult	Large	Low	Medium
SEC	Short	Simple	Medium	High	Medium
IAC	Short	Simple	Small	Medium	Highest
PP	Short	Simplest	Large	Medium	Low
MF	Short	Medium	Very small	High	High

* Qualitative descriptors are based on comparative studies. Actual performance depends on sample type and protocol.

## Data Availability

Not applicable.

## Abbreviations

The following abbreviations are used in this manuscript:

EVs	Extracellular vesicles
ESCRT	Endosomal sorting complexes required for transport
BBB	Blood–brain barrier
UC	Differential ultracentrifugation
SEC	Size exclusion chromatography
IAC	Immunoaffinity capture
PP	Polymer precipitation
MF	Microfluidic
ISEV	International Society for Extracellular Vesicles
FDA	Food and Drug Administration
